# Cleavable Cross-Linkers Redefined by a Novel MS^3^-Trigger Algorithm

**DOI:** 10.1021/acs.analchem.3c01673

**Published:** 2023-10-10

**Authors:** Lars Kolbowski, Lutz Fischer, Juri Rappsilber

**Affiliations:** † Technische Universität Berlin, Chair of Bioanalytics, 10623 Berlin, Germany; ‡ Wellcome Centre for Cell Biology, University of Edinburgh, Edinburgh EH9 3BF, United Kingdom; § Si-M/“Der Simulierte Mensch”, a Science Framework of Technische Universität Berlin and Charité - Universitätsmedizin Berlin, 10623 Berlin, Germany

## Abstract

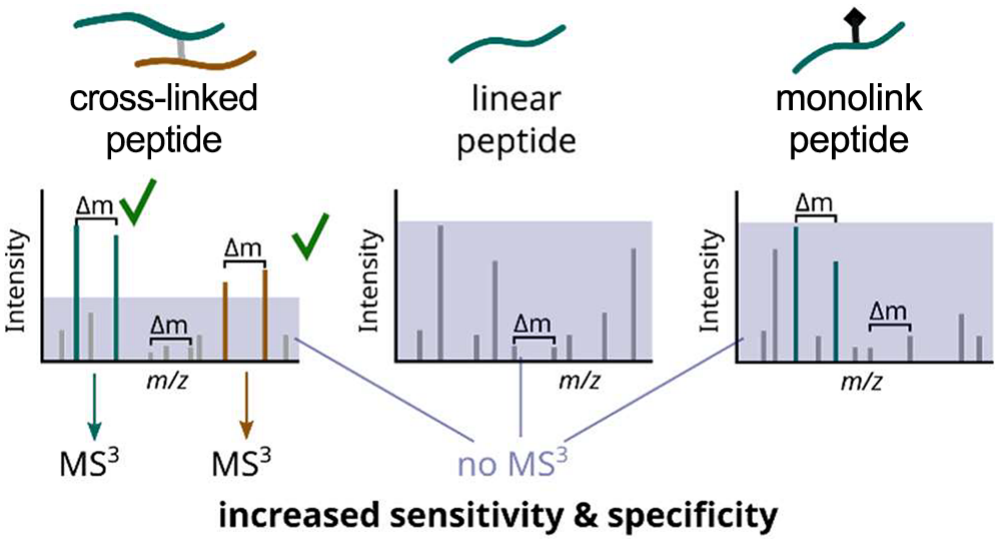

Cross-linking mass spectrometry (MS) is currently transitioning from a routine tool in structural biology to enabling structural systems biology. MS-cleavable cross-linkers could substantially reduce the associated search space expansion by allowing a MS^3^-based approach for identifying cross-linked peptides. However, MS^2^ (MS/MS)-based approaches currently outperform approaches utilizing MS^3^. We show here that the sensitivity and specificity of triggering MS^3^ have been hampered algorithmically. Our four-step MS^3^-trigger algorithm greatly outperformed currently employed methods and comes close to reaching the theoretical limit.

Cross-linking mass spectrometry (cross-linking MS) is a discovery tool of hitherto hidden aspects of biology,^1^ from placing protein sequence in unassigned densities of cryoEM and cryoET data^2^ to capturing weak interactions in whole cells that are lost upon lysis.^3^ Among the plethora of cross-linking MS workflows,^4−6^ the use of MS-cleavable cross-linkers stands out.^7−12^ Their cleavage reverses the cross-link in the mass spectrometer, such that the two peptides can be analyzed individually. While this can also be achieved computationally during data analysis,^13^ doing so in the mass spectrometer improves peptide fragmentation.^14^ Accordingly, cleavable cross-linkers improve the number of reliably identifiable cross-links, especially in complex samples.^14,15^ In principle, the unlinked peptides could also be selected for separate fragmentation in MS^3^.^16^ MS^3^ would tremendously simplify data analysis; however, most investigations focus on MS^2^ (MS/MS) data.^17,18^ The additional acquisition time cost due to the acquisition of MS^3^ spectra is one of the downsides of MS^3^-based approaches^14,19^ which would need to be counterbalanced by clear advantages. However, MS^3^ approaches based on MS-cleavable cross-linkers are currently limited by two technical challenges:^14^ while the signature doublets of the two peptides can routinely be detected in the spectra of cross-linked peptides (81%), only 41% of the individual peptides are selected for MS^3^ due to low sensitivity. In addition, much time is wasted on MS^3^ of non-cross-linked peptides that are present at higher abundance in the analyzed samples (i.e., low specificity). This is the problem with current algorithms used for MS^3^ decision making, and we present here a novel algorithm for MS^3^-based acquisition strategies.

We first designed a four-step procedure that we felt was highly likely to improve results based on our previous analysis.^20^ First, isotopic envelopes in the spectrum are detected, deconvoluted to give monoisotopic peaks with summed intensities, and assigned charge states (Figure 1a). Second, for each peak with a defined charge state, its theoretical doublet partner peak is calculated by the addition of the doublet delta mass divided by the charge state. These theoretical doublet peaks are then matched against peaks with matching charge states, considering a user-defined relative *m*/*z* tolerance (Figure 1b). Third, a rank-based cutoff is applied, and all doublets with a rank smaller than 20 are disregarded, to reduce noise matching (Figure 1c). Fourth, we implemented a “second peptide mass” filter, eliminating doublets that leave less than 500 Da for the second peptide. This results in a final matched-doublet list (Figure 1d). If the remaining mass is less than 500 Da, no MS^3^ should be acquired because the doublet stems either from a linear peptide with a cross-linker modification, is a false-positive random match, or the second peptide is too small to be reliably identifiable (Figure S1). We chose 5 ppm as the mass tolerance for matching the doublet delta mass, based on accuracy of the data and balancing specificity and selectivity (Figure S2).

**Figure 1:**
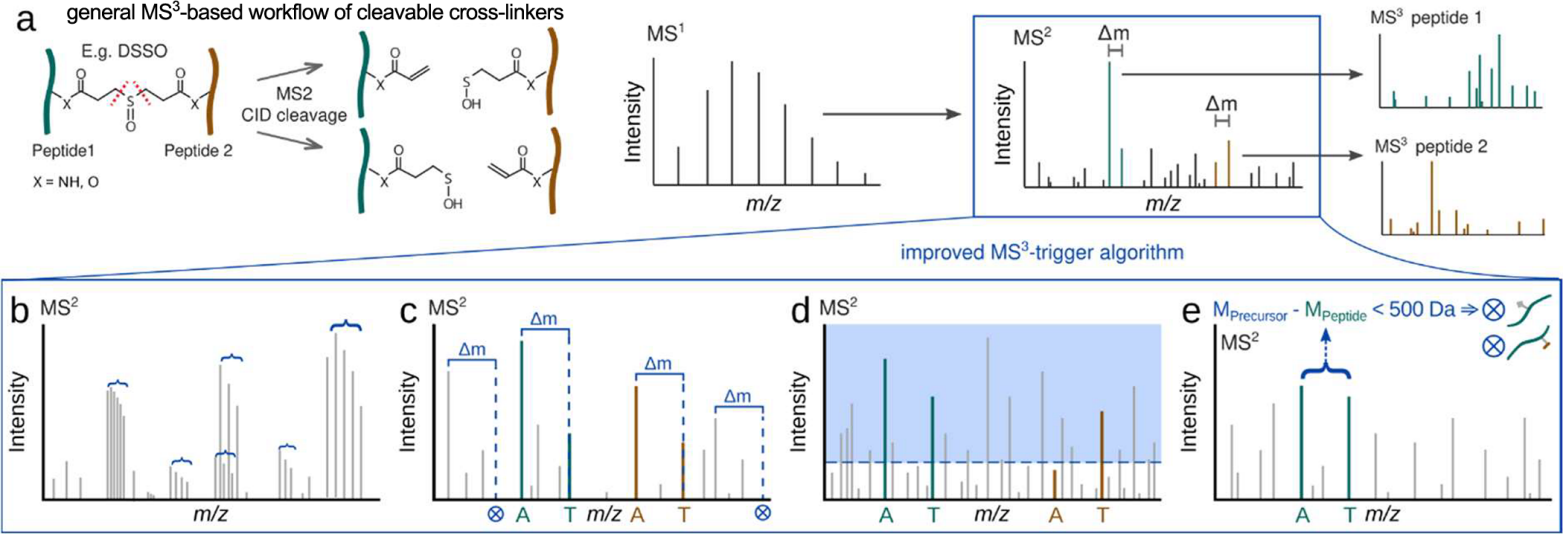
Improved MS^3^-trigger algorithm for MS^2^ spectra. (a) General MS^3^-based workflow of MS-cleavable cross-linkers. MS^3^ of individual peptides is triggered based on MS^2^-cleavage of the cross-linked peptide pair, using doublet signals resulting from the asymmetric cross-linker cleavage (adapted from O’Reilly and Rappsilber^4^). (b–e) Flowchart of the improved MS^3^-trigger algorithm for MS^2^ spectra. (b) Deconvolution of isotope clusters and charge determination. (c) Doublet matching by Δ*m* and charge state. (d) Doublets must have at least one doublet peak among the top 20 peaks ranked by intensity (intensity rank filter). (e) The mass difference between a doublet and the precursor mass is required to be >500 Da (second peptide mass filter). This excludes linear peptides that are modified with a cross-linker and therefore lead to doublets in MS^2^. The filter also excludes cross-linked peptides with a second peptide that is too short for reliable identification.

Next, we tested the algorithm on two publicly available data sets that utilized MS^3^-based acquisition methods, the Ribosome^21^ and Synaptosome^22^ data sets. Each matched doublet from the final output of our algorithm would have triggered the acquisition of a MS^3^ spectrum. We evaluated this against MS^3^ actually triggered during the acquisition with regard to specificity and sensitivity. With respect to sensitivity, for the best-case scenario, we would expect to trigger an MS^3^ for a doublet of every peptide that we found by annotating the identified cross-link-spectrum matches (CSMs). This gives a theoretically possible maximum of 85% of all CSMs for the Synaptosome data set (Figure 2a) and 76% for the Ribosome data set (Figure 2d). The rate of correctly triggered doublets improved on both peptide doublets from 58% to 79% and from 37% to 60% of CSMs for the Synaptosome and Ribosome data sets, respectively, thus approaching the theoretical maximum (Figure 2b, e). In addition, our doublet selection algorithm vastly improved the specificity in both data sets (Figure 2c, f). We were able to almost completely eliminate MS^3^ spectra triggered by linear peptide–spectrum matches (PSMs) (−92%). On average, only 0.11 and 0.09 of MS^3^ spectra were triggered per MS^2^ spectrum in the Synaptosome and Ribosome data sets, respectively, by these non-cross-linked peptides. In addition, MS^3^ spectra triggered by cross-linker-modified linear peptides were also reduced substantially, by approximately 75% on average.

**Figure 2:**
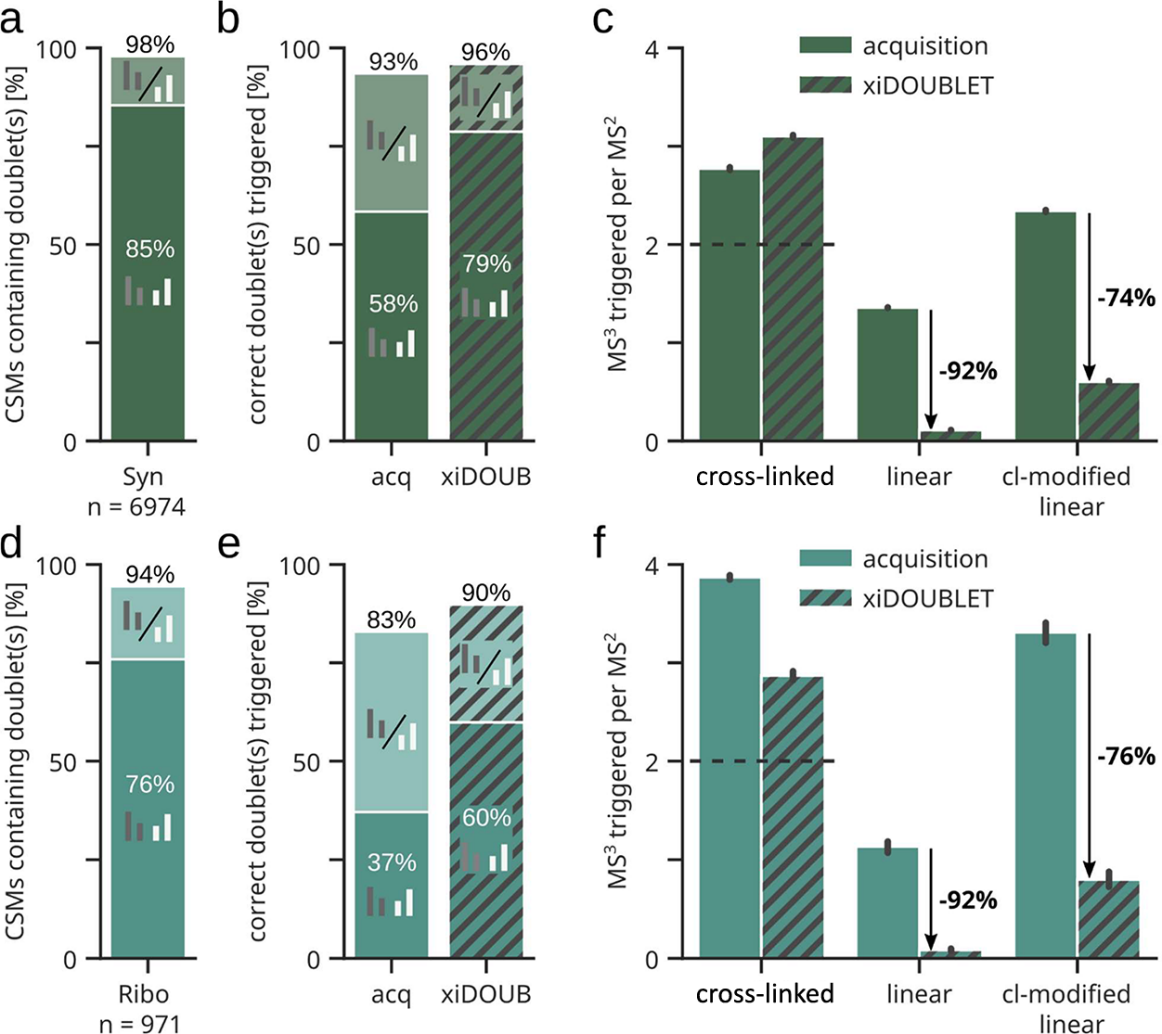
Sensitivity and specificity of MS^3^ triggering. (a, d) Proportion of identified cross-linked-spectrum matches (CSMs) that contain one (lighter colors) or both (darker colors) peptide doublets in each data set (5% CSM-level FDR). (b, e) Proportion of correctly triggered MS^3^ scans, comparing data from acquisitions to the results of the xiDOUBLET algorithm. (c, f) Number of triggered MS^3^ scans per MS^2^ scan, comparing data from acquisitions to the xiDOUBLET algorithm. Ideally, two MS^3^ scans are triggered for a cross-link (one for each of the two peptides, dotted line) and none for linear and modified linear peptides. Error bars show the 0.95 confidence interval. Panels (a–c) represent the Synaptosome, panels (d–f) the Ribosome data.

Gains in sensitivity and time during acquisition in addition to gains in selectivity greatly affect the depth of the analysis. We have demonstrated here that sensitivity and specificity can be improved considerably with no change in the experimental design of established cross-linking MS protocols. Our four-step procedure vastly outperforms the doublet selection algorithm currently employed on Orbitrap mass spectrometers. The closed source code of the instrument control software prevents us from implementing our procedure on Orbitraps, however, making a case for open-source code in the interest of scientific progress. We therefore extend an urgent call to the vendor to implement the improved procedure on Orbitraps. Our work shows, nonetheless, that MS^3^ approaches suffer from algorithmic restrictions that can be overcome and that overcoming these restrictions will help to unfold the full potential of MS-cleavable cross-linkers for structural proteomics. Once these improvements are implemented, it will be interesting to repeat direct comparisons with stepped-HCD MS2, the currently best performing acquisition strategy for cross-linked peptides.^20,21^

## Experimental Section

We reanalyzed the publicly available CID MS^3^-based data from the Ribosome data set (PXD011861) and Synaptosome (PXD010317 and PXD015160) data set. The data sets were analyzed as previously described.^14^ A 5% CSM level FDR, with sequence-consecutive and minimum peptide length (5 amino acids) filters, was applied. The CID spectra were annotated using pyXiAnnotator according to the CSM identification. We annotated the b- and y-ion series and the cleavable cross-linker stub fragments A, S, and T using a 15 ppm fragment mass tolerance. For the doublet rank evaluation, the “deisotoped max rank” column from the pyXiAnnotator output (which determines the rank of the annotated isotope cluster by comparing the maximum intensity peak of each isotope cluster) was used. A doublet rank was then assigned based on the higher of the two doublet peak ranks. To determine if the correct peaks were being triggered for MS^3^, the MS^3^ precursor *m*/*z* was extracted from the scan header of the MS^3^ spectra associated with the unique CSMs passing FDR (as described above) and compared with the corresponding MS^2^-CID annotation result. If the MS^3^ precursor matched a cross-linked peptide stub fragment within 20 ppm error tolerance, it was assigned as “correctly triggered”. For the evaluation of the MS^3^ trigger specificity, the number of MS^3^ scans associated with nonunique CSMs and linear PSMs (with and without hydrolyzed or amidated cross-linker modifications) which were above the FDR threshold was used.

The settings for the xiDOUBLET doublet detection algorithm used here were ms2_tol of 5 ppm tolerance, cross-linker DSSO, stubs A & T, rank_cutoff of 20, cap of 4, and second_peptide_mass_filter 500. The algorithm is written in Python and is open source and freely available on https://github.com/Rappsilber-Laboratory/xiDOUBLET.

## Supporting Information Available

Distribution of second peptide masses (Figure S1) and sensitivity and specificity of MS^3^ triggering using different mass tolerances (Figure S2) (PDF)
