# Supplementary material for: Cleavable Cross-Linkers Redefined by a Novel MS3-Trigger Algorithm
Source: Anal Chem. 2023 Oct 10;95(42):15461–4. doi: 10.1021/acs.analchem.3c01673 (PMC10603603; doi:10.1021/acs.analchem.3c01673)
Supplement: Supplementary file 1 — ac3c01673_si_001.pdf [file ac3c01673_si_001.pdf]

Supporting Information for

Cleavable crosslinkers redefined by a novel MS^3-trigger algorithm

Lars Kolbowski[1], Lutz Fischer[1], Juri Rappsilber[1,2,3]

[1] Technische Universität Berlin, Chair of Bioanalytics, 10623 Berlin, Germany
[2] Wellcome Centre for Cell Biology, University of Edinburgh, Edinburgh EH9 3BF, UK
[3] Si-M/"Der Simulierte Mensch", a Science Framework of Technische Universität Berlin and Charité - Universitätsmedizin Berlin, 10623 Berlin, Germany.

Correspondence to juri.rappsilber@tu-berlin.de

This file includes:

Supplementary Figures S1-2

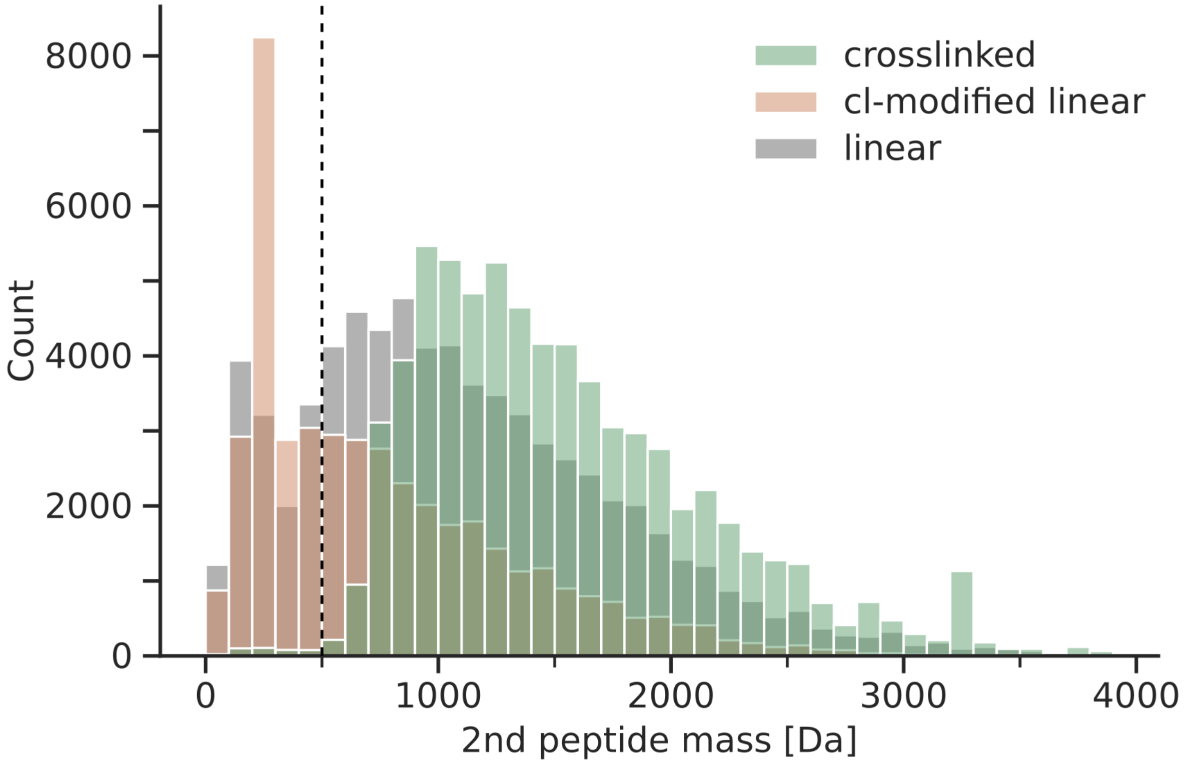

Figure S1. Distribution of second peptide masses. Histogram of remaining second peptide masses for all doublets (filtered to top 20 peaks and capped to the max. 4 highest ranking doublets per spectrum) detected in identified CSMs (green) and linear PSMs with crosslinker modification (orange) and without (grey) from both datasets. The dashed line shows the chosen cut-off.

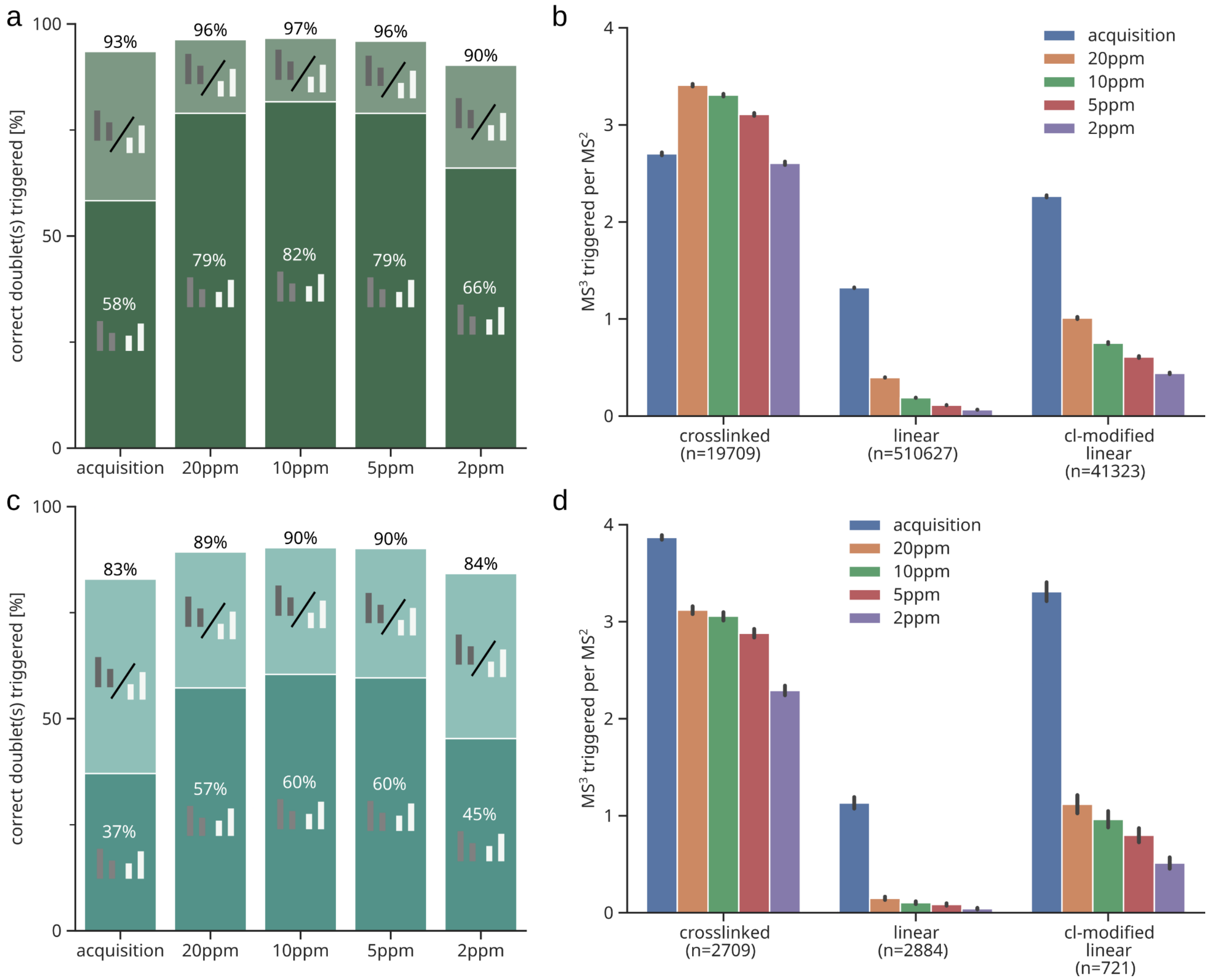

Figure S2. Sensitivity and specificity of MS^3 triggering using different mass tolerances. (a, c) Proportion of correctly triggered MS^3 scans acquisition data compared to the results from using in-silico triggering using the xiDOUBLET algorithm. (b, d) Number of triggered MS^3 scans per MS^2 scan, comparing DSSO acquisition results to the xiDOUBLET algorithm. Error bars show the 0.95 confidence interval. Panels (a - b) represent the Synaptosome, panels (c - d) the Ribosome data.
